# Etiology and Epidemiology of Travelers’ Diarrhea among US Military and Adult Travelers, 2018–2023

**DOI:** 10.3201/eid3014.240308

**Published:** 2024-11

**Authors:** Melissa S. Anderson, Evelyn W. Mahugu, Hayley R. Ashbaugh, Aaron G. Wellbrock, Maia Nozadze, Sanjaya K. Shrestha, Giselle M. Soto, Rania A. Nada, Prativa Pandey, Mathew D. Esona, Daniel J. Crouch, Michelle Hartman-Lane, Hunter J. Smith

**Affiliations:** Naval Health Research Center, San Diego, California, USA (M.S. Anderson, A.G. Wellbrock, M.D. Esona, D.J. Crouch, M. Hartman-Lane); General Dynamics Information Technology, San Diego (M.S. Anderson, A.G. Wellbrock); Armed Forces Health Surveillance Division, Silver Spring, Maryland, USA (E.W. Mahugu, H.J. Smith); General Dynamics, Silver Spring (E.W. Mahugu); US Food and Drug Administration, Silver Spring (H.R. Ashbaugh); Walter Reed Army Institute of Research Europe-Middle East, Tbilisi, Georgia (M. Nozadze); Walter Reed Armed Forces Research Institute of Medical Sciences Research Unit Nepal, Kathmandu, Nepal (S.K. Shrestha); US Naval Medical Research Unit SOUTH, Lima, Peru (G.M. Soto); US Naval Medical Research Unit EURAFCENT, Cairo, Egypt (R.A. Nada); Canadian International Water and Energy Consultants Clinic, Kathmandu (P. Pandey).

**Keywords:** Travelers’ diarrhea, acute diarrhea, acute gastroenteritis, epidemiology, etiology, military, Escherichia coli, Salmonella, Shigella, Campylobacter, norovirus, bacteria, antimicrobial resistance, travel, United States

## Abstract

Travelers’ diarrhea has a high incidence rate among deployed US military personnel and can hinder operational readiness. The Global Travelers’ Diarrhea study is a US Department of Defense­–funded multisite surveillance effort to investigate the etiology and epidemiology of travelers’ diarrhea. During 2018–2023, we enrolled 512 participants at partner institutions in 6 countries: Djibouti, Georgia, Egypt, Honduras, Nepal, and Peru. Harmonized laboratory methods conducted at each partner institution identified >1 pathogens, including *Escherichia coli* (67%–82%), norovirus (4%–29%), and *Campylobacter jejuni* (2%–20%), in 403 (79%) cases. Among cases, 79.7% were single infections, 19.6% were double infections, and 0.7% were triple infections. The most common enterotoxigenic *E. coli* colonization factors identified were CS3 (25%) and CS21 (25%), followed by CS2 (18%) and CS6 (15%). These data can inform best treatment practices for travelers’ diarrhea and support US military health readiness.

Travelers’ diarrhea (TD) is a gastrointestinal (GI) illness that affects millions of people each year, and infection rates range from 30% to 70% among travelers within 2 weeks of travel initiation, depending upon geographic region and seasonality of travel (*1*,*2*). Symptoms can range from mild cramps and loose stool to bloody diarrhea, fever, abdominal pain, and vomiting. Bacterial pathogens are the leading causative agents of TD, accounting for >80% of cases (*1*).

In March 2023, the United States Military Infectious Disease Research Panel’s Threat Prioritization Panel determined that bacterial diarrhea was the number 1 infectious disease threat to US military operations (*3*). In 2013, the Global Emerging Infections Surveillance branch, in collaboration with its worldwide network of partner laboratories and the Naval Health Research Center’s Operational Infectious Diseases Directorate, launched the Global Travelers’ Diarrhea (GTD) study to address issues posed to the US military by TD (*4*,*5*). This article describes the epidemiology of TD cases among US military populations and adult travelers during 2018–2023. In addition, this article characterizes coinfections, bacterial virulence factors, and pathogen factors relevant for medical countermeasure development.

## Material and Methods

### Partner Institutions and Enrollment Sites

**Figure 1:**
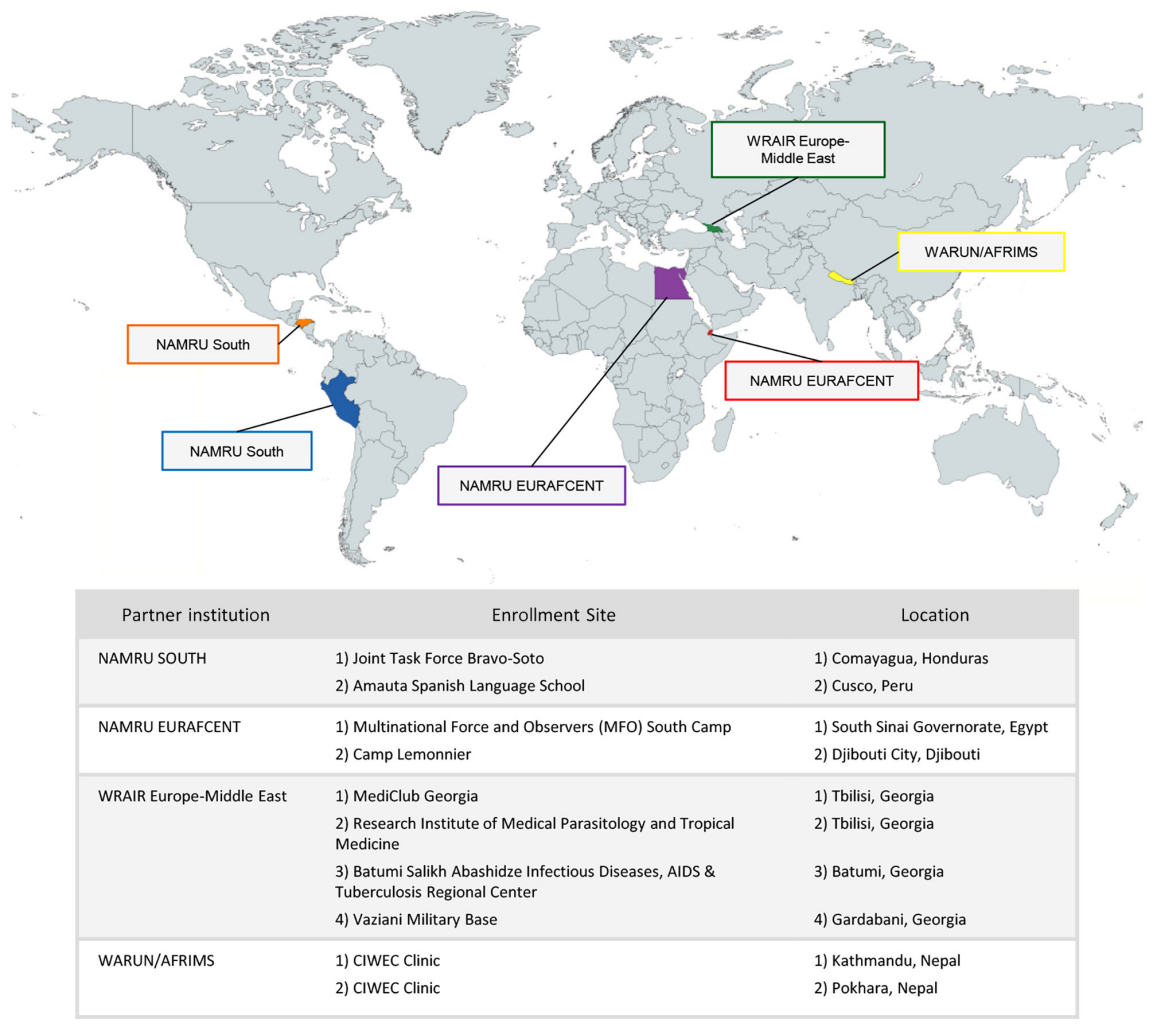
Partner institutions and enrollment site locations for a study of etiology and epidemiology of travelers’ diarrhea among US military personnel and adult travelers, 2018–2023. The US Naval Medical Research Unit (NAMRU) south enrollment sites: orange, Comayagua, Honduras; blue, Lima, Peru. The US Naval Medical Research Unit EURAFCENT detachment enrollment sites: purple, Cairo, Egypt; red, Djibouti City, Djibouti. Walter Reed Army Institute of Research (WRAIR) Europe-Middle East enrollment sites: green, Tbilisi, Batumi, and Gardabani, Republic of Georgia. Walter Reed Armed Forces Research Institute of Medical Sciences Research Unit Nepal (WARUN/AFRIMS) enrollment sites: yellow, Kathmandu and Pokhara, Nepal.

Partner institutions included the Walter Reed Armed Forces Research Institute of Medical Sciences Research Unit Nepal located in Kathmandu, Nepal, which had enrollment sites in Kathmandu and Pokhara, Nepal; US Naval Medical Research Unit EURAFCENT detachment in Cairo, Egypt, which had enrollment sites in South Sinai Governorate, Egypt, and Djibouti City, Djibouti; US Naval Medical Research Unit SOUTH located in Lima, Peru, which had enrollment sites in Cusco, Peru, and Comayagua, Honduras; and Walter Reed Army Institute of Research Europe-Middle East located in Tbilisi, Georgia, which had enrollment sites in Tbilisi, Batumi, and Gardabani, Georgia (Figure 1).

### Participant Enrollment

Each partner institution was required to have institutional review board approval before collecting GTD Study–associated samples. Upon determination of eligibility, participants signed an informed consent agreement, except at the US Naval Medical Research Unit EURAFCENT; its local institutional review board did not require informed consent after January 2022. Study participation was voluntary. The study period was October 2018–April 2023.

### Inclusion and Exclusion Criteria

The study inclusion criteria were adult travelers, >18 years of age, originating from the United States or any other upper-middle or high-income country (Appendix Table 1) (*6*), who had been in the enrollment country for <1 year, seeking healthcare services for acute GI illness. We defined illness as either acute diarrhea or acute gastroenteritis beginning >3 days after home departure. Adult travelers for this study included US military personnel, government employees, and citizens (e.g., nongovernmental organization workers, tourists, students, etc.). Patients were excluded from the study if they were experiencing chronic, persistent GI symptoms of >7 days before enrollment or noninfectious diarrhea or could not produce a fecal sample.

### Case 

The GTD study defined acute diarrhea as >3 loose/liquid feces, or >2 loose/liquid feces plus >2 additional GI symptoms. Case definitions for acute gastroenteritis were >3 vomiting episodes plus >1 additional GI symptom, or >2 vomiting episodes plus >2 additional GI symptoms occurring within the past 24 hours. Additional GI symptoms include diarrhea, vomiting, nausea, flatulence, cramping, muscle aches, headache, decreased urination, loss of appetite, bloating, abdominal pain, joint aches, malaise, fatigue, fever, or bloody feces.

### Questionnaire

We assisted participants with completing a structured, standardized questionnaire. The questionnaire included questions about data variables describing demographics, symptoms, travel history, disposition, functional abilities, and treatment received.

### Sample Receiving and Processing

Fecal specimens were stored as raw feces, in Cary-Blair (CB) medium, or feces in Cary-Blair with indicator (CBI) transport medium at 4°C for a maximum of 48 hours before transportation at 4°C to the GTD partner laboratory to perform testing. The partner laboratory assessed fecal specimens to verify appropriate temperature. Specimens that were frozen or room temperature were discarded and excluded from the study. Specimens received as raw feces were processed to a 20% weight/volume solution in phosphate-buffered saline before downstream testing, and specimens received in CB or CBI transport medium were directly used for downstream testing. Samples were either tested immediately upon arrival or frozen at –80°C and batch tested later.

### Nucleic Acid Extraction and PCR

We extracted total nucleic acid from 20% fecal suspensions, fecal suspensions in CB or CBI, or boil prepped bacterial suspensions by using the QIAamp Viral RNA Mini kit (QIAGEN, https://www.qiagen.com). We used a total nucleic acid template for real-time PCR (rPCR) assays. We chose gene targets based on previously published literature (*7**–**10*).

We conducted real-time reverse transcription PCR (rRT-PCR) specific for norovirus genogroup I and II (GI and GII) by using the AgPath One Step RT-PCR kit (Ambion-Thermo Fisher Scientific, https://www.thermofisher.com) (*7*). We conducted rPCR specific for bacteria to detect the following organisms: *E. coli* pathotypes including enterotoxigenic *E. coli* (ETEC), enteroaggregative *E. coli* (EAEC), enteropathogenic *E. coli* (EPEC), shiga-like toxin­–producing *E. coli* (STEC), and enteroinvasive *E. coli* (EIEC)/*Shigella* (*8*,*9*); *Campylobacter jejuni*, including subspecies *jejuni* and *doylei* (*10*); and *Salmonella enterica* ssp. *enterica* (*10*). We conducted rPCR specific for ETEC colonization factors on total nucleic acid extracted from isolated colonies (*9*). We used the PerfeCTa qPCR Tough Mix Kit (Quantabio, https://www.quantabio.com) for DNA amplification in all bacterial PCR assays. Primer and probe sequences, PCR conditions, and multiplex assay details are available (Appendix Tables 2, 3). We conducted all PCR reactions by using the Applied Biosystems 7500 Fast Dx or 7500 Fast Real-Time PCR instruments (Thermo Fisher Scientific, https://www.thermofisher.com).

**Table 2:** Positivity and pathogen coinfections recovered from participants, by geographic region and country, in a study of etiology and epidemiology of travelers’ diarrhea among US military personnel and adult travelers, 2018–2023*

Sample positivity	Geographic region and country										Total, n = 512
	South and Central America			Northern Africa		Sub-Saharan Africa		Southern Asia		Eastern Europe	
	Honduras, n = 107	Peru, n = 15		Egypt, n = 17		Djibouti, n = 200		Nepal, n = 133		Georgia, n = 40	
Positive samples†	80 (75)	12 (80)		15 (88)		146 (73)		119 (89)		31 (78)	403 (79)
Single infections	71 (89)	7 (58)		15 (100)		130 (89)		75 (63)		23 (74)	321 (80)
Double infections	8 (10)	5 (42)		0		15 (10)		43 (36)		8 (26)	79 (20)
Triple infections	1 (1)	0		0		1 (1)		1 (1)		0	3 (1)
Total no. pathogens‡	178	26		28		288		276		71	867

*Values are no. (%), except as indicated. Percentages are of total positive samples, not samples tested. Samples cannot be positive for both Shiga-like toxin–producing *Escherichia coli* and enteropathogenic *E. coli* because the *eae* gene used to identify enteropathogenic *E. coli* can be found in Shiga-like toxin–producing *E. coli*; therefore, samples that tested positive for *stx*1/2 alone or in combination with *eae* were resulted as Shiga-like toxin–producing *E. coli*, and samples that tested positive for *eae* without *stx*1/2 were resulted as enteropathogenic *E. coli*. All other *E. coli* pathotype combinations were possible to identify. †Samples positive for >1 of the following pathogens: norovirus genogroup I, norovirus genogroup II, enteroaggregative *E. coli*, enterotoxigenic *E. coli*, enteropathogenic *E. coli*, Shiga-like toxin–producing *E. coli*, enteroinvasive *E. coli/Shigella*, *Salmonella*, and *Campylobacter*. ‡Only of pathogens tested for in this study.

**Table 3:** Enterotoxigenic *E. coli* colonization factors identified in pathogens recovered from participants of a study of etiology and epidemiology of travelers’ diarrhea among United States military personnel and adult travelers, by geographic region and country, 2018–2023*

Factors	Geographic region and country					Total, n = 106
	South/Central America		Sub-Saharan Africa		Southern Asia	
Honduras, n = 32	Djibouti, n = 37	Nepal, n = 37
CFA/I	3 (9)		0		0	3 (3)
CS4	0		2 (5)		2 (5)	4 (4)
CS6	3 (9)		7 (19)		6 (16)	16 (15)
CS14	0		1 (3)		1 (3)	2 (2)
CS1/PCF071	2 (6)		0		6 (16)	8 (8)
CS2	4 (13)		10 (27)		5 (14)	19 (18)
CS17/19	1 (3)		0		0	1 (1)
CS21	9 (28)		11 (30)		7 (19)	27 (25)
CS3	10 (31)		6 (16)		10 (27)	26 (25)
CS5	0		0		0	0
CS7	0		0		0	0
*Values are no. (%) enterotoxigenic *E. coli* samples from each country.						

### Bacteriology

We subcultured samples that were positive for ETEC by rPCR onto MacConkey agar and incubated them at 35–37°C for 18–24 hours (*11*). A laboratory testing schematic is available (Appendix Figure 1).

### Coinfection Analysis and Data Management

We analyzed coinfections by 4 major pathogen groups: norovirus (GI and GII), *E. coli* (EAEC, ETEC, EPEC, STEC, and EIEC/*Shigella*), *Salmonella*, and *Campylobacter*. We then cleaned the deidentified questionnaire and laboratory testing data by using Excel (Microsoft, https://www.microsoft.com) or Tableau Desktop version 2023.3 (Tableau, https://www.tableau.com). We merged questionnaire data and laboratory data in Excel by using participant identification numbers as the linking identifier.

## Results

During October 2018–April 2023, a total of 512 participants who met the acute diarrhea or acute gastroenteritis case definitions were enrolled in the GTD study in Honduras (21%), Peru (3%), Egypt (3%), Djibouti (39%), Nepal (26%), and Georgia (8%) (Table 1). The average participant age was 34 (SD 12) Years. Among participants, 35% were female, 59% male, and 6% unidentified sex. Participants were primarily born in North America (45%) or Europe (22%); however, that did not necessarily imply country of origin before travel and enrollment in the study. Most participants were US military service members (58%) or tourists (20%) (Table 1).

**Table 1:** Characteristics among acute diarrhea and acute gastroenteritis cases by geographic region and country, reported by participants in a study of etiology and epidemiology of travelers’ diarrhea among US military personnel and adult travelers, 2018–2023*

Characteristics	Geographic region and country										Total
	South and Central America			Northern Africa		Sub-Saharan Africa		Southern Asia		Eastern Europe	
	Honduras	Peru		Egypt		Djibouti		Nepal		Georgia	
Enrollments	107 (21)	15 (3)		17 (3)		200 (39)		133 (26)		40 (8)	512 (100)
Average age, y (SD)	34 (9)	NA		33 (6)		35 (11)		33 (14)		38 (16)	34 (12)
Unknown age	13 (12)	15 (100)		0		132 (66)		0		2 (5)	162 (32)
Sex											
F	26 (24)	10 (67)		3 (18)		38 (19)		84 (63)		17 (43)	178 (35)
M	81 (76)	5 (33)		14 (82)		131 (66)		49 (37)		23 (58)	303 (59)
Unknown	0	0		0		31 (16)		0		0	31 (6)
Birth region											
East Asia	0	0		0		0		6 (5)		0	6 (1)
North America	100 (93)	5 (33)		17 (100)		64 (32)		37 (28)		6 (15)	229 (45)
Europe	2 (2)	10 (67)		0		2 (1)		71 (53)		26 (65)	111 (22)
Oceania	0	0		0		0		11 (8)		0	11 (2)
Middle East	0	0		0		0		1 (1)		1 (3)	2 (<1)
Unknown	5 (5)	0		0		134 (67)		7 (5)		7 (18)	153 (30)
Traveler type											
US military	105 (98)	0		17 (100)		200 (100)		0		7 (18)	299 (58)
Government, US or non-US	2 (2)	0		0		0		0		2 (5)	4 (1)
Nongovernmental organization or aid worker	0	0		0		0		9 (7)		2 (5)	11 (2)
Tourist	0	0		0		0		85 (64)		19 (48)	104 (20)
Student	0	15 (100)		0		0		10 (8)		1 (3)	26 (5)
Other	0	0		0		0		29 (22)		2 (5)	31 (8)
Unknown	0	0		0		0		0		7 (18)	37 (6)

*Values are no. (%), except as indicated. Percentages may not total 100% because of rounding.

Across all sites, 403 (79%) of 512 samples tested positive for >1 pathogens, identifying a total of 867 pathogens (Table 2). Of the 403 positive samples, 79.7% were single infections, 19.6% were double infections, and 0.7% were triple infections. No samples were positive for all 4 pathogen groups (Table 2).

**Figure 2:**
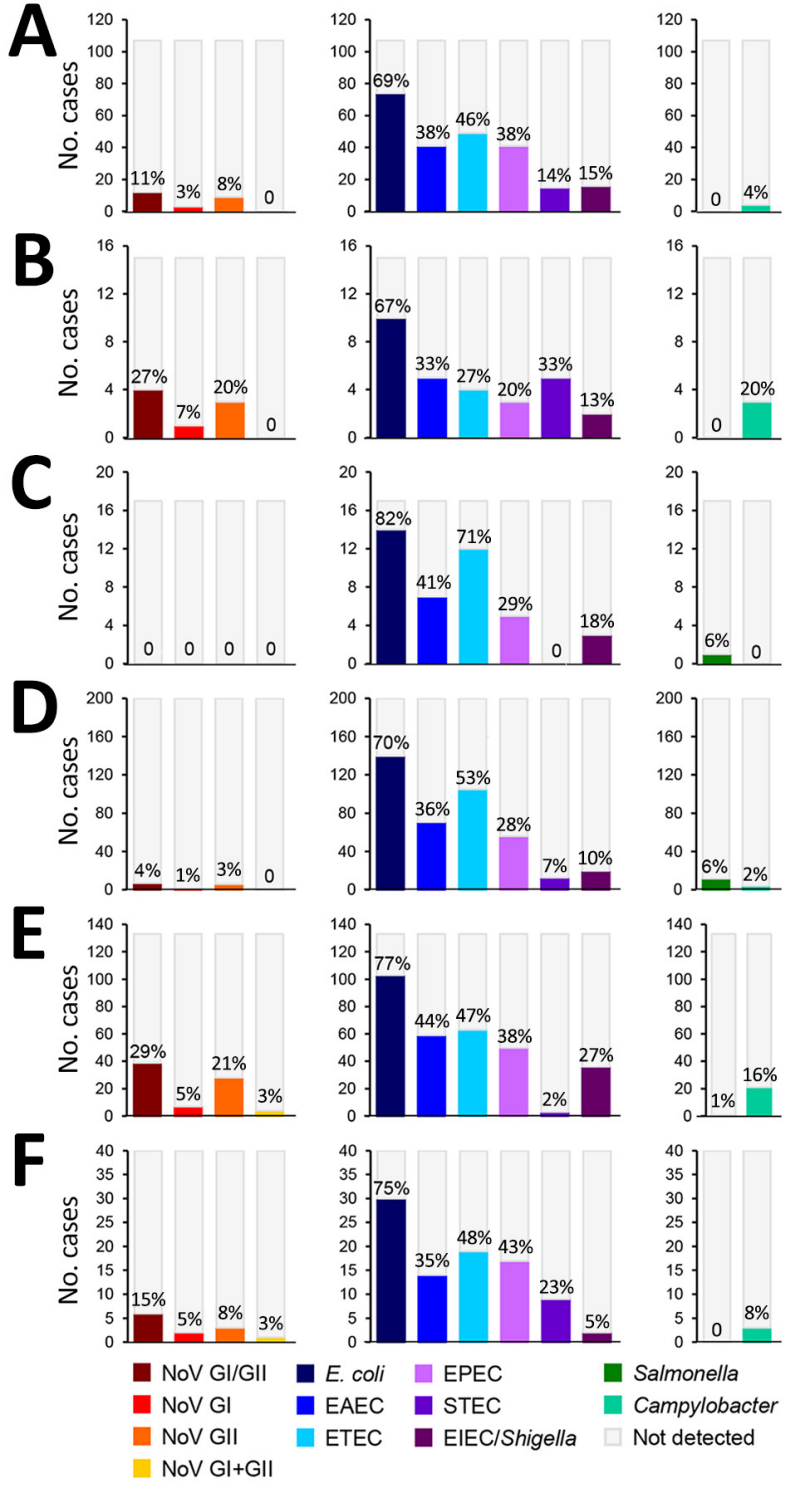
Enteric pathogens detected in a study of etiology and epidemiology of travelers’ diarrhea among US military personnel and adult travelers, 2018–2023. The study included travelers from the following countries: A) Honduras (n = 107); B) Peru (n = 15); C) Egypt (n = 17); D) Djibouti (n = 200); E) Nepal (n = 133); F) Georgia (n = 40). We consider the proportions from Peru, Egypt, and Georgia to be unstable (n < 40) and the results should be interpreted with caution. The y-axis and bars represent the number of times each pathogen was detected for each country. The percent positivity for each pathogen is listed above the corresponding bar in each graph. Percent positivity does not add up to 100% for each country because of the occurrence of coinfections. EAEC, enteroaggregative *Escherichia coli*; EIEC, enteroinvasive *E. coli*/*Shigella*; EPEC, enteropathogenic *E. coli*; ETEC, enterotoxigenic *E. coli*; g, genogroup; NoV, norovirus; STEC, Shiga-like toxin–producing *E. coli*.

*E. coli* was the most common pathogen identified in Peru (67%), Nepal (77%), Georgia (75%), Honduras (69%), Egypt (82%), and Djibouti (70%), whereas *Salmonella* was the least identified in all countries except Egypt (6%) and Djibouti (6%) (Figure 2). Coinfection analysis identified *E. coli* in a higher number of coinfections than any of the other 3 pathogen groups across all 6 countries (Appendix Figure 2).

ETEC colonization factors were identified from ETEC isolates recovered from samples collected in 3 countries: Honduras, Djibouti, and Nepal (Table 3). In total, 106 isolates were tested. The most identified ETEC colonization factors were CS3 (25%) and CS21 (25%), as well as CS2 (18%) and CS6 (15%) (Table 3). The least identified ETEC colonization factors were CS17/19 (1%) and CS14 (2%). Colonization factors CS5 and CS7 were not identified from any country (Table 3).

## Conclusions

This study describes the etiology and epidemiology of TD among US military and adult civilian travelers across South and Central America, Northern and sub-Saharan Africa, Southern Asia, and Eastern Europe. We found *E. coli* was the leading (67%–82%) etiology of TD across global surveillance sites (Figure 2). ETEC was the most identified *E. coli* pathotype in 5 of 6 countries (Figure 2). Our investigation also identified *Campylobacter*, *Salmonella*, and norovirus as TD etiologies, although with lower proportions than observed for *E. coli* (Figure 2). Those data on TD disease etiology are consistent with the literature discussing both military and civilian populations throughout the globe, indicating bacterial pathogens are the leading causative agents of disease (*4*,*12*–*15*). Our study data suggest *E. coli*, specifically pathotypes ETEC, EAEC, and EPEC, are the leading causes of TD in Southern and Central Asia, Northern Africa, the Middle East, sub-Saharan Africa, and Central and South America (Figure 2), which is consistent with the GTD study data for all 6 surveillance countries (*12*).

The highest rates of *Campylobacter* and *Salmonella* associated with TD are found in Southeast and East Asia, and high rates are also found in Southern and Central Asia (*12*). The GTD study did not include a surveillance site in Southeast or East Asia, but among included countries, we identified the highest rates of *Campylobacter* in Peru (20%) and Nepal (16%) and the highest rates of *Salmonella* in Egypt (6%) and Djibouti (6%) (Figure 2).

Previous work by the GTD study found *E. coli* (including all pathotypes tested for) in 42% of the cases enrolled during 2013–2018 (n = 410) (*4*), which is lower than the cases enrolled during 2018–2023 (n = 512; 72%) (Figure 2). That increase may represent improved laboratory diagnostic methods for *E. coli* pathotypes; samples collected during 2013–2018 were analyzed by conventional PCR, whereas samples collected during 2018–2023 were analyzed by rPCR. Of note, case numbers of *Salmonella* and *Campylobacter* were similar across study periods despite updates to laboratory protocols (Figure 2) (*4*).

Vaccines used prophylactically to prevent TD have the potential to reduce disease incidence and severity; however, no vaccines for *E. coli*, *Campylobacter*, or *Shigella* are currently licensed by the US Food and Drug Administration. ETEC vaccine candidates currently under investigation are based on antitoxin or anticolonization factor immunity. Approximately 50%–80% of all colonization factor-positive clinical ETEC isolates found within the general population encode colonization factors A/I, CS3, CS5, and CS6 (*16*), making them potential vaccine targets (*17*,*18*). In this study, CS6, CS3, CS2, and CS21 were the most identified colonization factors across the geographic regions tested; however, colonization factor A/I was only identified in 3% of isolates and CS5 was not identified in any isolate (Table 3). Our results combined with the efforts of the Global Emerging Infections Surveillance network of global surveillance laboratories, including maintaining repositories of clinical samples collected throughout the world, may help guide future vaccine development and therapeutics for TD and other diseases of interest to the US military and global health.

One limitation of this study is that we considered the disease etiology proportions from Egypt (n = 17), Peru (n = 15), and Georgia (n = 40) unstable because of low enrollment; we recommend interpreting the results with caution. In addition, selection bias may have influenced results because of site accessibility and the potential that travelers with mild TD are less likely to seek care than are travelers with severe TD, which might have affected the pathogens observed.

Moving forward, we recommend the GTD study expand to include antimicrobial resistance (AMR) characterization of bacterial pathogens identified from TD cases by using antimicrobial susceptibility testing and next-generation sequencing technologies to identify genetic markers of AMR and virulence factors of enteric bacterial pathogens. Those combined efforts could provide insight on the effects of AMR across unique global regions, enhance antimicrobial stewardship to limit changes in drug resistance patterns in enteric pathogens, and improve military health readiness through targeted prevention and treatment interventions for TD.

Appendix Additional information about etiology and epidemiology of travelers’ diarrhea among United States military and adult travelers, global traveler’s diarrhea study, 2018–2023.
