# Supplementary material for: Etiology and Epidemiology of Travelers’ Diarrhea among US Military and Adult Travelers, 2018–2023
Source: Emerg Infect Dis. 2024 Nov;30(Suppl 2):S19–25. doi: 10.3201/eid3014.240308 (PMC11559568; doi:10.3201/eid3014.240308)
Supplement: Appendix — Additional information about etiology and epidemiology of travelers’ diarrhea among United States military and adult travelers, global traveler’s diarrhea study, 2018–2023. [file 24-0308-Techapp-s1.pdf]

Article DOI: https://doi.org/10.3201/eid3014.240308

EID cannot ensure accessibility for supplementary materials supplied by authors. Readers who have difficulty accessing supplementary content should contact the authors for assistance.

Etiology and Epidemiology of Travelers' Diarrhea among US Military and Adult Travelers, 2018–2023

Appendix

References

1. Hidaka A, Hokyo T, Arikawa K, Fujihara S, Ogasawara J, Hase A, et al. Multiplex real-time PCR for exhaustive detection of diarrhoeagenic *Escherichia coli*. J Appl Microbiol. 2009;106:410–20. PubMed https://doi.org/10.1111/j.1365-2672.2008.04043.x

2. Liu J, Silapong S, Jeanwattanalert P, Lertsehtakarn P, Bodhidatta L, Swierczewski B, et al. Multiplex real time PCR panels to identify fourteen colonization factors of enterotoxigenic *Escherichia coli* (ETEC). PLoS One. 2017;12:e0176882. PubMed https://doi.org/10.1371/journal.pone.0176882

3. Wiemer D, Loderstaedt U, von Wulffen H, Priesnitz S, Fischer M, Tannich E, et al. Real-time multiplex PCR for simultaneous detection of *Campylobacter jejuni*, *Salmonella*, *Shigella* and *Yersinia* species in fecal samples. Int J Med Microbiol. 2011;301:577–84. PubMed https://doi.org/10.1016/j.ijmm.2011.06.001

Appendix Table 1. World Bank defined middle/upper-income and low-income countries

Country	World bank income classification
Afghanistan	Low Income
Albania	Mid-Upper/High Income
Algeria	Mid-Upper/High Income
Andorra	Low Income
Angola	Low Income
Antigua and Barbuda	Mid-Upper/High Income
Argentina	Mid-Upper/High Income
Armenia	Mid-Upper/High Income
Australia	Mid-Upper/High Income
Austria	Mid-Upper/High Income
Azerbaijan	Mid-Upper/High Income
Bahrain	Mid-Upper/High Income
Bangladesh	Low Income
Barbados	Mid-Upper/High Income
Belarus	Mid-Upper/High Income
Belgium	Mid-Upper/High Income
Belize	Mid-Upper/High Income

Country	World bank income classification
Benin	Low Income
Bhutan	Low Income
Bolivia	Low Income
Bosnia and Herzegovina	Mid-Upper/High Income
Botswana	Mid-Upper/High Income
Brazil	Mid-Upper/High Income
Brunei	Mid-Upper/High Income
Bulgaria	Mid-Upper/High Income
Burkina Faso	Low Income
Burundi	Low Income
Cabo Verde	Low Income
Cambodia	Low Income
Cameroon	Low Income
Canada	Mid-Upper/High Income
Central African Republic	Low Income
Chad	Low Income
Chile	Mid-Upper/High Income
People’s Republic of China	Mid-Upper/High Income
Colombia	Mid-Upper/High Income
Comoros	Low Income
Congo, Democratic Republic of the	Low Income
Congo, Republic of the	Low Income
Costa Rica	Mid-Upper/High Income
Côte d’Ivoire	Low Income
Croatia	Mid-Upper/High Income
Cuba	Low Income
Cyprus	Mid-Upper/High Income
Czech Republic	Mid-Upper/High Income
Denmark	Mid-Upper/High Income
Djibouti	Low Income
Dominica	Mid-Upper/High Income
Dominican Republic	Mid-Upper/High Income
East Timor (Timor-Leste)	Low Income
Ecuador	Mid-Upper/High Income
Egypt	Low Income
El Salvador	Low Income
England	Mid-Upper/High Income
Equatorial Guinea	Mid-Upper/High Income
Eritrea	Low Income
Estonia	Mid-Upper/High Income
Eswatini	Low Income
Ethiopia	Low Income
Fiji	Mid-Upper/High Income
Finland	Mid-Upper/High Income
France	Mid-Upper/High Income
Gabon	Mid-Upper/High Income
Georgia	Low Income
Germany	Mid-Upper/High Income
Ghana	Low Income
Greece	Mid-Upper/High Income
Grenada	Mid-Upper/High Income
Guatemala	Mid-Upper/High Income
Guinea	Low Income
Guinea-Bissau	Low Income
Guyana	Mid-Upper/High Income
Haiti	Low Income
Honduras	Low Income
Hungary	Mid-Upper/High Income
Iceland	Mid-Upper/High Income
India	Low Income
Indonesia	Low Income
Iran	Mid-Upper/High Income
Iraq	Mid-Upper/High Income
Ireland	Mid-Upper/High Income
Israel	Mid-Upper/High Income
Italy	Mid-Upper/High Income
Jamaica	Mid-Upper/High Income
Japan	Mid-Upper/High Income
Jordan	Mid-Upper/High Income

Country	World bank income classification
Kazakhstan	Mid-Upper/High Income
Kenya	Low Income
Kiribati	Low Income
North Korea	Low Income
Republic of Korea	Mid-Upper/High Income
South Korea	Mid-Upper/High Income
Kosovo	Low Income
Kuwait	Mid-Upper/High Income
Kyrgyzstan	Low Income
Lao People’s Democratic Republic	Low Income
Latvia	Mid-Upper/High Income
Lebanon	Mid-Upper/High Income
Lesotho	Low Income
Liberia	Low Income
Libya	Mid-Upper/High Income
Liechtenstein	Low Income
Lithuania	Mid-Upper/High Income
Luxembourg	Mid-Upper/High Income
Madagascar	Low Income
Malawi	Low Income
Malaysia	Mid-Upper/High Income
Maldives	Mid-Upper/High Income
Mali	Low Income
Malta	Mid-Upper/High Income
Marshall Islands	Mid-Upper/High Income
Mauritania	Low Income
Mauritius	Mid-Upper/High Income
Mexico	Mid-Upper/High Income
Micronesia, Federated States of	Low Income
Moldova	Low Income
Monaco	Low Income
Mongolia	Low Income
Montenegro	Mid-Upper/High Income
Morocco	Low Income
Mozambique	Low Income
Myanmar (Burma)	Low Income
Namibia	Mid-Upper/High Income
Nauru	Mid-Upper/High Income
Nepal	Low Income
Netherlands	Mid-Upper/High Income
New Zealand	Mid-Upper/High Income
Nicaragua	Low Income
Niger	Low Income
Nigeria	Low Income
North Macedonia	Mid-Upper/High Income
Norway	Mid-Upper/High Income
Oman	Mid-Upper/High Income
Pakistan	Low Income
Palau	Mid-Upper/High Income
Panama	Mid-Upper/High Income
Papua New Guinea	Low Income
Paraguay	Mid-Upper/High Income
Peru	Mid-Upper/High Income
Philippines	Low Income
Poland	Mid-Upper/High Income
Portugal	Mid-Upper/High Income
Qatar	Mid-Upper/High Income
Romania	Mid-Upper/High Income
Russia	Mid-Upper/High Income
Rwanda	Low Income
Saint Kitts and Nevis	Mid-Upper/High Income
Saint Lucia	Mid-Upper/High Income
Saint Vincent and the Grenadines	Mid-Upper/High Income
Samoa	Mid-Upper/High Income
San Marino	Low Income
Sao Tome and Principe	Low Income
Saudi Arabia	Mid-Upper/High Income
Senegal	Low Income
Serbia	Mid-Upper/High Income

Country	World bank income classification
Seychelles	Mid-Upper/High Income
Scotland	Mid-Upper/High Income
Sierra Leone	Low Income
Singapore	Mid-Upper/High Income
Slovakia	Mid-Upper/High Income
Slovenia	Mid-Upper/High Income
Solomon Islands	Low Income
Somalia	Low Income
South Africa	Mid-Upper/High Income
Spain	Mid-Upper/High Income
Sri Lanka	Low Income
Sudan	Low Income
Sudan, South	Low Income
Suriname	Mid-Upper/High Income
Sweden	Mid-Upper/High Income
Switzerland	Mid-Upper/High Income
Syria	Low Income
Taiwan	Low Income
Tajikistan	Low Income
Tanzania	Low Income
Thailand	Mid-Upper/High Income
The Bahamas	Mid-Upper/High Income
The Gambia	Low Income
Togo	Low Income
Tonga	Mid-Upper/High Income
Trinidad and Tobago	Mid-Upper/High Income
Tunisia	Low Income
Turkey	Mid-Upper/High Income
Turkmenistan	Mid-Upper/High Income
Tuvalu	Mid-Upper/High Income
Uganda	Low Income
Ukraine	Low Income
United Arab Emirates	Mid-Upper/High Income
UK	Mid-Upper/High Income
United Kingdom	Mid-Upper/High Income
USA	Mid-Upper/High Income
United States	Mid-Upper/High Income
Uruguay	Mid-Upper/High Income
Uzbekistan	Low Income
Vanuatu	Low Income
Vatican City	Low Income
Venezuela	Low Income
Vietnam	Low Income
Yemen	Low Income
Zambia	Low Income
Zimbabwe	Low Income

Appendix Table 2. Primer and probe sequences

Pathogen	Assay	Primer/probe name	Primer set	Target	Sequence (5′ → 3′)
Pathogenic *E. coli*	Assay 1 ETEC	NA	HLT (*1*)	*elt*	HLT-F – TTC CCA CCG GAT CAC CAA
		NA			HLT-R – CAA CCT TGT GGT GCA TGA TGA
		NA			HLT-Pro – /NED/ CTT GGA GAG AAG AAC CCT /MGB/
		NA	STh (*2*)	*estH*	STh-F – GCT AAA CCA GYA GRG TCT TCA AAA
		NA			STh-R – CCC GGT ACA RGC AGG ATT ACA ACA
		NA			STh-Pro – /VIC/ TGG TCC TGA AAG CAT GAA /MGB/
		NA	STp (*2*)	*estP*	STp-F – TGA ATC ACT TGA CTC TTC AAA A
		NA			STp-R – GGC AGG ATT ACA ACA AAG TT
		NA			STp-Pro – /FAM/ TGA ACA ACA CAT TTT ACT GCT /MGB/
	Assay 2 EAEC, EIEC/Shigella	NA	EIEC (*1*)	*virB*	virB-F – GGA TTT GTG CAA CGA CTT GTT AAG
		NA			virB-R – GAG GAA TCT TGG CTT TGA TAA AGG
		NA			virB-Pro – /VIC/ CAC TCC ATT CTG GTA ATA A /MGB/
		NA	EAEC (*1*)	*aggR*	aggR-333f – CAG CGA TAC ATT AAG ACG CCT AAA G
		NA			aggR-448r – CGT CAG CAT CAG CTA CAA TTA TTC C
		NA			aggR-pro – /FAM/ CTT GCA GTT GTC CGA ATT /MGB/
	Assay 3 EPEC, STEC	NA	EPEC (*1*)	*eae*	eae-F – CCG ATT CCT CTG GTG ACG A
		NA			eae-R – CCA CGG TTT ATC AAA CTG ATA ACG
		NA			eae-Pro – /NED/ (Cy3) CGT CAT GGT ACG GGT AA /MGB/
		NA	Shiga Toxin 1 (*1*)	*stx1*	Stx1-F – ACT TCT CGA CTG CAA AGA CGT ATG
		NA			Stx1-R – ACA AAT TAT CCC CTG AGC CAC TAT C
		NA			Stx1-Pro – /FAM/ CTC TGC AAT AGG TAC TCC A /MGB/
		NA	Shiga Toxin 2 (*1*)	*stx2*	Stx2-F – CCA CAT CGG TGT CTG TTA TTA ACC
		NA			Stx2-R – GGT CAA AAC GCG CCT GAT AG
		NA			Stx2-Pro – /VIC/ TTG CTG TGG ATA TAC GAG G /MGB/
Salmonella enterica and *Campylobacter jejuni*	NA	NA	Salmonella (*3*)	*ttrR*	F – AAT TAG CCA TGT TGT AAT CTC
		NA			R – ATT GTT GAT TCA GGT ACA AAC
		NA			P - /JOE/ CAA GTT CAA CGC GCA ATT TA /BHQ1/
		NA	Campylobacter (*3*)	*gyrA*	F – CTA TAA CAA CTG CAC CTA CTA AT
		NA			R – ATG AAA TTT TTG CCA GTG GTG
		NA			P - /FAM/ CTT AAT AGC CGT CAC CCC AC /BHQ1/
		NA	GFP*	*gfp77*	F – AGA TGA CGG GAA CTA CAA G
		NA			R – CCT TCA GCT CGA TTC TAT T
		NA			P - /Cy5/ CAC CTT CGA ACT TGA CTT CAG CGC RQSp/
Norovirus, Genogroup					
I	NA	Cog 1F	NA	NA	CGY TGG ATG CGI TTY CAT GA
I	NA	Cog 1R	NA	NA	CTT AGA CGC CAT CAT CAT TYA C
II	NA	Cog 2F	NA	NA	CAR GAR BCN ATG TTY AGR TGG ATG AG
II	NA	Cog 2R	NA	NA	TCG ACG CCA TCT TCA TTC ACA
NA	NA	MS2-F	NA	NA	TGG CAC TAC CCC TCT CCG TAT TCA CG
NA	NA	MS2-R	NA	NA	GTA CGG GCG ACC CCA CGA TGA C
I	NA	Ring 1C	NA	NA	56-FAM – TGG ACA GGR GAY CGC – MGBNFQ
II	NA	Ring 2	NA	NA	Cy5 – TGG GAG GGC GAT CGC AAT CT – 3BHQ_2
NA	NA	MS2-P	NA	NA	Cy3 – CAC ATC GAT AGA TCA AGG TGC CTA CAA GC – 3IAbRQSp
ETEC Colonization Factors	Assay 1 ETEC CF	NA	CFA/I (*1*)	*cfaB*	AGCTTATTCTCCCGCATCAAA
		NA			GAACATCTGTAAGCTGTGGTGT
		NA			/FAM/ ACTCAAGTACATACAAACGATGCA /BHQ1/
		NA	CS4 (*1*)	*csaB*	CTATTCACCTGCGGCAAGTC
		NA			GGGGAGTTGTTTTGTAGAATCCA
		NA			/TexRD/ TCGCAACTAAAGTTCATACAAATGT /IBFQ/
		NA	CS6 (*1*)	*cssB*	GGAGTGGTAAATGCAGGAAACT
		NA			GAACAGCGGAATCAATATCTGGA
		NA			/JOE/ CTCTGGATGTAAATGTAAATATTGAG /IBFQ/
		NA	CS14 (*1*)	*csuA1*	TCATGGGCAGGGAAGACATT
		NA			TACTATTCGAAACACCTGCCG
		NA			/Cy5/ AGTTGGCGATCTGGGTTTTG /IBFQ/
	Assay 2 ETEC CF	NA	CS1/PCF071 (*1*)	*csoA*	ACTTTGCTTCGAGTGGTGTT
		NA			CCCTGATATTGACCAGCTGTTA
		NA			/FAM/ CAGAAACTTTCAATCCATGCAGAT/BHQ1/
		NA	CS2 (*1*)	*cotA*	TCTGCTCGTATCAATACCCAAGTT
		NA			GTGCCAGCGAATGAAACCTCTA
		NA			/TexRd-XN/ TCTGATCCAAGCAAGACTATTCC/IBFQ/
		NA	CS17/19 (*1*)	*csbA-csdA*	AGGSAGTTGTAGTGAAGCTGT
		NA			GTCACTTTCATCGGAATTTGCGA
		NA			/JOE/ CAGTTCTGTCCAATATTATGAAGCCA /IBFQ/

Pathogen	Assay	Primer/probe name	Primer set	Target	Sequence (5′ → 3′)
		NA	CS21 (*1*)	*lngA*	GGACCCATTAAGCCTTACTGC
		NA			GTTATTACGCACTTCGTCTGGT
		NA			/5Cy5/TGCAGCACAGTTAGTTCAGC /IBFQ/
	Assay 3 ETEC CF	NA	CS3 (*1*)	*cstA*	GGTCTTTCACTGTCAGCTATGA
		NA			CCAAGTTGCATCCAGAGCTG
		NA			/FAM/ TGGCATTAAATGTGCTTTCTCCT /BHQ1/
		NA	CS5 (*1*)	*csfA*	GCGTGACACGTCAGCTAATATAAAC
		NA			AAAGTGATTGCGACTTCCCC
		NA			/5Cy5/ ACCGCAGTAGAAGCAGCTAA /IBFQ/
		NA	CS7 (*1*)	*csvA*	TGCTCCCGTTACTAAAAATACGT
		NA			CGAACGGGCTGTGATACCTT
		NA			/TexRd-XN/ CCAATCCGTTCACAAAAGCC /IBFQ/

*NHRC in-house assay; IBFQ, Iowa Black Fluorescence Quencher

Appendix Table 3. Real-time PCR conditions*

Pathogen	Cycle	Condition
Pathogenic *E. coli*	1	95°C for 10 min
	45	95°C for 15 s
	45	60°C for 1 min
Salmonella enterica and *Campylobacter jejuni*	1	95°C for 10 min
	45	95°C for 15 s
	45	60°C for 1 min
Norovirus	1	45°C for 10 min
	1	95°C for 10 min
	40	95°C for 15 s
	40	60°C for 1 min
ETEC Colonization Factors	1	95°C for 10 min
	45	95°C for 15 s
	45	60°C for 1 min

*Fluorescence data should be collected during the 60°C incubation step.

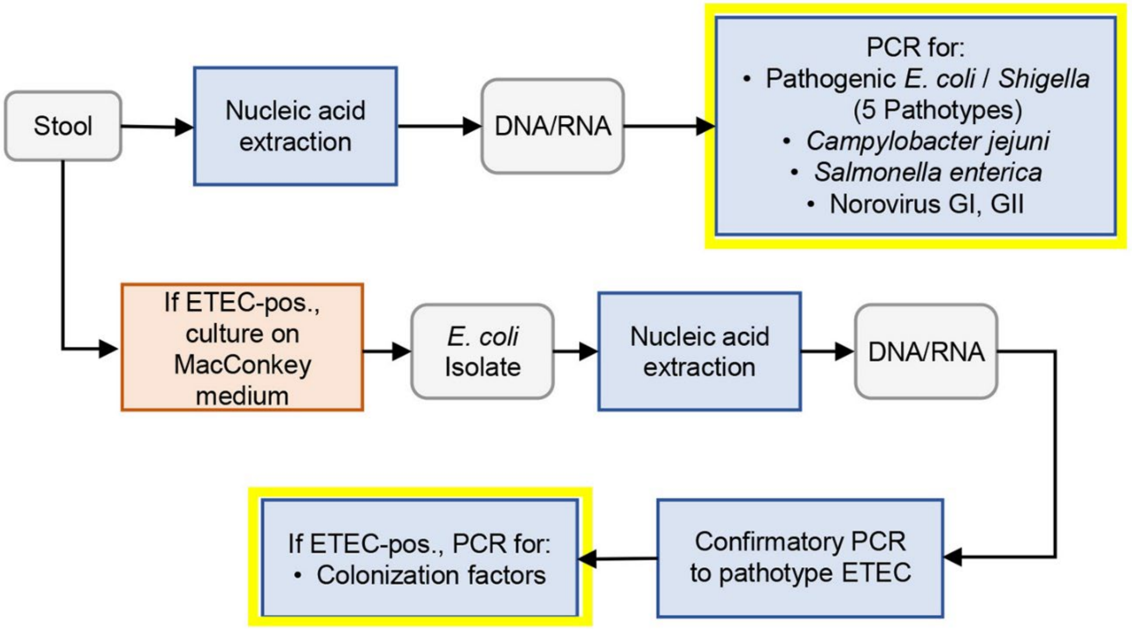

Appendix Figure 1. Laboratory testing schematic. Laboratory workflow for all GTD Study samples. Gray: Biologic material. Orange: Microbiology workflow. Blue: Molecular workflow. Boxes outlined in yellow signify data presented in this study.

Co-infection count

Honduras

	NoV GI/GII	PEC	*Salmonella*	*Campylobacter*
NoV GI/GII	**5**			
PEC	7	**65**		
Salmonella	0	0	**0**	
Campylobacter	1	3	0	**1**

Peru

	NoV GI/GII	PEC	*Salmonella*	*Campylobacter*
NoV GI/GII	**1**			
PEC	3	**5**		
Salmonella	0	0	**0**	
Campylobacter	0	2	0	**1**

Egypt

	NoV GI/GII	PEC	*Salmonella*	*Campylobacter*
NoV GI/GII	**0**			
PEC	0	**14**		
Salmonella	0	0	**1**	
Campylobacter	0	0	0	**0**

Djibouti

	NoV GI/GII	PEC	*Salmonella*	*Campylobacter*
NoV GI/GII	**1**			
PEC	6	**124**		
Salmonella	0	8	**4**	
Campylobacter	0	3	1	**1**

Nepal

	NoV GI/GII	PEC	*Salmonella*	*Campylobacter*
NoV GI/GII	**10**			
PEC	29	**59**		
Salmonella	0	1	**0**	
Campylobacter	1	15	0	**6**

Georgia

	NoV GI/GII	PEC	*Salmonella*	*Campylobacter*
NoV GI/GII	**0**			
PEC	6	**22**		
Salmonella	0	0	**0**	
Campylobacter	0	2	0	**1**

Appendix Figure 2. Coinfection counts by country. Coinfection counts of NoV (including NoV GI and NoV GII), *E. coli* (including EAEC, ETEC, EPEC, STEC, and EIEC/*Shigella*), *Salmonella*, and *Campylobacter*. Bold numbers (shown in the diagonal from top left to bottom right) represent single infections.
